# Uptake of hormonal contraceptives and correlates of uptake in a phase III clinical trial in rural South Western Uganda

**DOI:** 10.1186/s12978-017-0296-3

**Published:** 2017-03-11

**Authors:** Andrew Abaasa, Mitzy Gafos, Zacchaeus Anywaine, Andrew Nunn, Angela Crook, Jonathan Levin, Sheena McCormack, Anatoli Kamali

**Affiliations:** 1 MRC/UVRI Uganda Research Unit on AIDS, P.O Box 49, Entebbe, Uganda; 2 MRC Clinical Trials Unit at UCL, London, UK; 3 Faculty of Health Sciences, School of Public Health, University of the Witwatersrand, Johannesburg, South Africa

**Keywords:** Hormonal contraceptives, Contraceptive uptake, Microbicides, Clinical trials

## Abstract

**Background:**

Use of a reliable contraception method has become an inclusion criterion in prevention trials to minimize time off product. We report on hormonal contraceptive prevalence, uptake, sustained use and correlates of use in the Microbicides Development Programme (MDP 301) trial at the Masaka Centre in Uganda.

**Methods:**

HIV negative women in sero-discordant relationships were enrolled and followed-up for 52 to 104 weeks from 2005 to 2009. Contraceptive use data was collected through self-report at baseline and dispensing records during follow-up. Hormonal contraceptives were promoted and provided to women that were not using a reliable method at enrolment. Baseline contraceptive prevalence, uptake and sustained use were calculated. Uptake was defined as a participant who reported not using a reliable method at enrolment and started using a hormonal method at any time after. Logistic regression models were fitted to investigate predictors of hormonal contraceptive uptake.

**Results:**

A total of 840 women were enrolled of whom 21 aged ≥50 years and 12 without follow-up data were excluded; leaving 807 (median age 31 IQR 26–38) in this analysis. At baseline, 228 (28%) reported using a reliable contraceptive; 197 hormonal, 28 female-sterilisation, two IUCD and one hysterectomy. As such 579 were not using a reliable contraceptive at enrolment, of whom 296 (51%) subsequently started using a hormonal contraceptive method; 253 DMPA, four oral pills, and two norplant. Overall 193 (98%) existing users and 262 (88%) new users sustained use throughout follow-up. Independent correlates of hormonal contraceptive uptake were: younger women ≤30 years, aOR = 2.5, 95% CI: 1.7–3.6 and reporting not using contraceptives at baseline due to lack of access or money, breastfeeding or other reasons, in comparison to women who reported using unreliable method.

**Conclusion:**

Promotion and provision of hormonal contraception doubled the proportion of women using a reliable method of contraception. Uptake was pronounced among younger women and those not previously using a reliable method because of lack of access or money, and breastfeeding. Promotion and provision of hormonal contraceptives in trials that require the interruption or discontinuation of investigational products during pregnancy is important to reduce the time off product.

**Trial registration:**

Protocol Number ISRCTN64716212.

## Plain English Summary

The use of reliable contraceptives is increasingly being added as an inclusion criterion in HIV prevention trials. Studies have not reported on the uptake of reliable contraceptives after enrolment among women participating in large phase III microbicide clinical trials to inform future trials. In this article we determined uptake of hormonal contraceptives and its correlates in a phase III vaginal microbicide trial.

Hormonal contraceptives (pill, injectable Depot medroxyprogesterone acetate (DMPA) and Norplant or intrauterine contraceptive device (IUCD)) were promoted and provided to women that were not using a reliable method at enrolment into the trial and during follow up.

Data on 807 women were analysed and 579 were not using a reliable contraceptive method at enrolment, of which 296 started using a hormonal contraceptive method mainly DMPA (253).

Promotion and provision of hormonal contraceptives substantially improved uptake of these methods. The uptake was particularly highest among younger women and among women who couldn’t or didn’t think they needed to access contraceptives. This evidence highlights the benefit of promoting and providing hormonal contraceptives to trial participants where pregnancy could disrupt the use of investigational products.

## Background

In 2014 it was estimated that only 28% [1] of women in Africa and 17% [2] in Sub-Saharan Africa were using modern methods of contraception (injectable, norplant, intrauterine contraceptive device (IUCD), pill, diaphragm, male condom and sterilization). In Uganda, modern contraceptive use among women of reproductive age increased from 18% in 2006 [3] to 32% in 2011 [4]. The proportion using modern methods is lower than that reported in neighbouring Kenya (39%) and Rwanda (45%), similar to Tanzania (26%) but higher than Burundi (18%) in 2011 [5]. Generally, the use of modern contraceptives in Uganda is lower among women aged 25 years or less (20%), married (26%), or those living in rural areas (23%) [3] [6]. The unmet need for any contraceptives in Uganda remains high at 41% especially among women who are currently married, living in rural areas and living in the Northern region [7]. In 2011, the total fertility rate among women of reproductive age in Uganda was estimated to be 6.2 and as a consequence the country’s population is expected to double over the next 20 years [5]. Addressing the unmet contraceptive needs of Ugandan women is critical for couples to limit or space births [8].

In addition, the need for female initiated and controlled HIV preventive options remains high. In 2011, the HIV prevalence among women of reproductive age in Uganda was 8% [9] and incidence of approximately 1% [10]. Contraceptive use among sero-discordant couples is low 23% [11] and similar to the national average among married women in general [12]. Similarly, condom use among sero-discordant couples is equally low (36%) [11]. Dual contraceptive use (condoms plus a modern contraceptive) among HIV positive women of reproductive age attending urban clinics is not good either 12%, with 42% of HIV-positive pregnant women reporting their pregnancy to be unintended [13]. Overall, condom use with a spouse or partner is low among men (6%) and women (4%) [14]. However, condom use while having sex with causal sexual partners has increased and it is still more likely among men than women [14].

A number of clinical trials evaluating new HIV prevention technologies including vaginal microbicides and oral pre-exposure prophylaxis (PrEP) have been conducted in Uganda [15–19]. These trials are powered to provide sufficient sample sizes to assess the efficacy of investigational products. Given the unknown effects of the investigational products on the unborn foetus, product use is usually interrupted or discontinued during pregnancy. A higher than anticipated pregnancy incidence may affect the statistical power of a trial to detect the efficacy of a product. Limiting the risk of unintended pregnancies in clinical trials by supporting women’s use of reliable contraceptives (injectable, norplant, pill, IUCD and female sterilisation) helps to avoid a loss of statistical power. The use of reliable contraceptives is increasingly being added as an inclusion criterion in HIV prevention trials [20]. The use of contraceptives at time of enrolment in later stage microbicide clinical trials has ranged from 10% in Nigeria [21] to 56% in Uganda [15] and South Africa [22]. The baseline use of hormonal (injectable, norplant and pills) contraceptives has ranged from 9% in Nigeria [21] to 15% in Ghana [23]. To date, studies have not reported on the uptake of reliable contraceptives after enrolment among women participating in large phase III microbicide clinical trials. Microbicide trials enrol women who are not intending to get pregnant during the course of the trial, yet pregnancy rates in efficacy trials have ranged from 1 to 27 per 100 women-years [24].

In this analysis we report on the baseline contraceptive use, correlates of hormonal contraceptive uptake after enrolment, and the sustained use of hormonal contraceptives during follow-up among women in sero-discordant couple sexual relationship enrolled in the Microbicides Development Programme (MDP 301) clinical trial in South-Western Uganda.

## Methods

MDP301 was an international, randomized, double-blind, placebo-controlled parallel-group phase III clinical trial, designed to evaluate the safety and efficacy of 0.5% and 2% PRO2000 candidate microbicide gels in preventing vaginally acquired HIV-1 infection. The trial design and trial results have been reported elsewhere [15, 25, 26]. In summary, participants were enrolled at 13 clinics across six research centres, three in South Africa and one each in Tanzania, Uganda and Zambia. This analysis is based on MDP301 data collected exclusively at the Masaka clinic research centre in South-Western Uganda.

The MDP301 Masaka clinical trial centre enrolled HIV-negative healthy women in a known HIV sero-discordant relationship and followed them for a minimum of 52 weeks and a maximum of 104 weeks. Women in sero-discordant relationships were identified and enrolled following a sero-survey conducted between September 2005 and August 2008. Follow-up continued until September 2009. The eligibility criteria are in Table 1.

**Table 1:** MDP 301 Uganda: eligibility criteria

Eligible	Ineligible
• Sexually active • 16 years old or above • HIV-negative at screening • Willing to undergo regular HIV testing and receive the result before randomisation • Willing to undergo regular speculum examinations and genital infection screens • Willing to have regular urine pregnancy tests • Willing to use study gel as instructed • Willing to receive health education about condoms • Willing and able to give informed consent	• Unable or unwilling to provide a reliable method of contact for the field team • Likely to move permanently out of the area within the next year • Likely to have sex more than 14 times a week on a regular basis during the course of follow-up • Using spermicides regularly • Pregnant or within 6 weeks postpartum at enrolment • Had a severe clinical or laboratory abnormality • Requiring referral for assessment of a clinically suspicious cervical lesion • Had treatment to the cervix, or to the womb through the cervix, within 30 days of enrolment • Had known latex allergy • Participating, or having participated within 30 days of enrolment, in a clinical trial of an unlicensed product, microbicide, barrier method or any other intervention likely to impact on the outcome of this trial ● Considered unlikely to be able to comply with the protocol

Details of the clinical, laboratory and pharmacy procedures, data management, field activities, counselling package and follow-up schedules are described elsewhere [15, 25–27]. Women were randomised to one of the three gel groups; 0.5% PRO2000, 2% PRO2000 or placebo. Screening visits occurred no more than 6 weeks prior to enrolment, and follow-up visits were scheduled every four weeks after enrolment and conducted at either the research clinic or designated government health centres. At screening, data were collected on demographic and behavioural characteristics. The following variables were considered in this analysis: age, religion, education level, employment status, method of contraceptives used and reason for non-use. Behavioural data were collected at the four weekly follow-up visits, including data on gel and condom use at the last sex act. Extended behavioural data were collected at the longer clinical examination visits, which occurred at weeks 4, 12, 24, 40, 52, 64, 76, 88, 100 and 104 after enrolment. Contraceptive use data were collected at every visit. Women were asked whether they were using any contraceptive method, if they were, a single method was captured which could include unreliable methods such as condoms, the rhythm method, withdrawal or traditional remedies. If they reported not using a contraceptive method, a single reason for none-use was captured which could include wanting to become pregnant, the partner being sterilised, or currently breastfeeding (locally considered a period of infertility). However, women who reported using an unreliable method were not asked why they were not using a reliable method.

The research team offered contraceptive services at every visit, which included the provision of the oral contraceptive pill and injectable Depot medroxyprogesterone acetate (DMPA). The study clinic referred women who chose to use the norplant or intrauterine contraceptive device (IUCD) to the Marie Stopes clinic located about one km from the study clinic. Women were given contraceptive cards, which captured the woman’s contraceptive use history and future prescription renewal dates. They were asked to bring their contraceptive cards at every visit. At each four-weekly follow-up visit we conducted rapid urine pregnancy tests and interrupted gel if the woman tested positive for pregnancy. The study provided transport expenses to and from the study clinic.

Enrolled women ranged in age from 16 to 59 years old. Only women of reproductive age between 16 and 49 years were included in this analysis. We further excluded women that did not have any follow-up data. We defined reliable contraceptives as non-barrier methods likely to significantly reduce the risk of pregnancy and categorised the following contraceptive methods as reliable: female sterilisation, pill, DMPA, Norplant and IUCD. We included the one woman who had had a hysterectomy in the group defined as using reliable contraceptive methods. We categorised condoms, the rhythm method, withdrawal, traditional remedies, breastfeeding or no method as ‘not reliable’. We did not classify condom use as a reliable method of contraception because in this community only male condom is readily and freely available and its use is largely male controlled. Women who were not using a reliable contraceptive at baseline were encouraged to use hormonal methods (pill, DMPA, or Norplant). We categorised ‘new’ hormonal contraceptive users as any women who reported not using a reliable contraceptive method at the enrolment visit and reported using a hormonal method during at least one follow-up visit.

### Statistical methods

The analysis was separated into three parts, first comparing women who reported using a reliable contraceptive method at enrolment to women who reported not using a reliable method. Secondly, women who started using a hormonal method after enrolment (new users) were compared to those that did not switch to a hormonal method (non-adopters) during the trial. Lastly, hormonal users that sustained use of the initial method overtime were compared to those that switched between hormonal methods after initiation. We defined sustained use of a hormonal method as a woman taking up a hormonal method and using it throughout the study follow up period.

Descriptive statistics were used to summarise women’s characteristics. The proportion of women using hormonal contraceptive was estimated as the number using hormonal contraceptives divided by the number of women attending the visit. We examined associations between women using reliable and those not using reliable contraceptives at baseline, and between women who started and sustained use of a hormonal method to those that switched between hormonal methods using chi-square. The associations between new hormonal contraceptive users and non-adopters during follow up were assessed using odds ratios, with 95% confidence intervals (CI), and by fitting logistic regression models. Only factors for which the association attained a statistical significance at the 15% [28] level using a likelihood ratio test (LRT) in a univariable analysis were considered for the multivariable model. In the multivariable model, factors were removed from the model using a backward elimination algorithm if removing the term did not make the fit of the model significantly worse at the 5% level on a likelihood ratio test (LRT). All analyses were conducted using Stata 11 (StataCorp, College Station, Texas, USA).

## Results

**Fig. 1:**
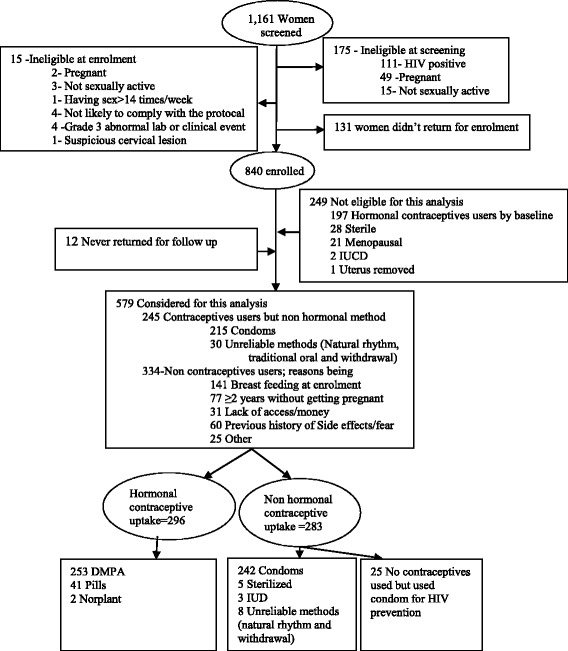
Masaka Centre study profile

A total of 1,161 women were screened and 840 (72.4%) enrolled in Uganda. The main reasons for ineligibility were HIV-positive status (111), pregnancy (51), not being sexually active (18), clinical findings making enrolment inadvisable (5), being unlikely to comply with the protocol (4) or likely to have sex more than 14 times per week (1) Fig. 1. An additional 131 women were eligible at screening but chose not to enrol in the study. Of the 840 women enrolled, 33 were excluded from this analysis, 21 women aged 50 or above and 12 women that did not have any follow up data. This left 807 women for analysis. The median age of women included in the analyses was 31 years (inter-quartile range IQR: 26–38).

### Contraceptive use at baseline

At baseline, 228 women (28.2%) reported using a reliable contraceptive method. A further 245 women (30.4%) reported using an unreliable contraceptive method, mainly condoms (215), the natural rhythm method (15), traditional oral methods (14) and withdrawal (1); 334 women (41.4%) reported not using any method of contraception.

Of the 228 women using a reliable contraceptive method, 197 (24.4% of the 807 women) reported using hormonal contraceptives (161 using DMPA, 33 using the oral pill and 3 using Norplant), 28 (3.5%) were sterilised, two (0.2%) were using the IUCD, and one (0.1%) had had a total hysterectomy. The only factor significantly associated with use of a reliable contraceptive method at enrolment was employment status with women who were in full time employment or who were housewives being more likely to use reliable contraception than unemployed women (Table 2).

**Table 2:** Baseline characteristics of reliable contraceptive users

Characteristic	N (col %)	Using a reliable contraceptive n (row %)	P-value
All participants	807	228 (39.4)	
Median age years (IQR)	31 (26-38)	30 (25-36)	0.106
Age group(years)			
31+	415 (51.4)	104 (25.1)	0.106
25-30	240 (29.8)	74 (30.8)	
16 – 24	152 (18.8)	50 (32.9)	
Religion			
Christians	701 (86.9)	200 (28.5)	0.652
Muslim	106 (13.1)	28 (26.4)	
Level of education			
None	117 (14.5)	26 (22.2)	0.258
Primary	548 (67.9)	158 (28.8)	
Secondary+	142 (17.6)	44 (31.0)	
Employment status			
Employed full time	99 (12.3)	36 (36.4)	0.025
Unemployed	592 (73.3)	152 (25.7)	
House wife	116 (14.4)	40 (34.5)	
Condom use at the last sex act in the last 4 weeks prior to enrolment			
No	240 (29.7)	70 (29.2)	0.725
Yes	508 (63.0)	144 (28.3)	
Did not have sex	59 (7.3)	14 (23.7)	

Col %); Column percentage

### Uptake of hormonal contraceptives

Table 3 presents the characteristics of the 579 women who were not using a reliable method of contraception at baseline. The median age was 31 (IQR: 26–38) with 51.4% being more than 30 years old. The majority were Christian (86.9%), had attained only primary education (67.9%) and were unemployed (73.3%).

**Table 3:** Factors associated with uptake of hormonal contraceptive methods in multivariable model

Characteristic	N (col %)	Up take of Hormonal contraceptives n (row %)	uOR (95% CI)	LRT P-value	aOR (95% CI)
All participants	579	296 (51.1)			
Age group in years					
31+	311 (53.7)	116/311 (37.3)	1	<0.001	1
16 – 30	268 (46.3)	180/268 (67.2)	3.3 (2.4-4.7)		2.5 (1.7-3.6)
Religion^*^					
Christians	503 (86.9)	251/503 (49.9)	1	0.122	
Muslim	76 (13.1)	45/76 (59.2)	1.5 (0.9-2.4)		
Level of education					
None	92 (15.9)	43/92 (46.7)	1	0.747	
Primary	390 (67.4)	204/390 (52.3)	1.2 (0.8-1.9)		
Secondary+	97 (16.7)	49/97 (50.5)	1.1 (0.6-2.0)		
Employment status					
Employed full time	62 (10.7)	26/62 (41.9)	1	0.150	
Unemployed	440 (76.0)	225/440 (51.1)	1.5 (0.9-2.6)		
House wife	77 (13.3)	45/77 (58.4)	1.8 (0.9-3.5)		
Contraceptive method at enrolment					
Condom	215 (37.1)	85/215 (39.5)	1	<0.001	
No method	334 (57.7)	204/334 (61.1)	2.4 (1.7-3.4)		
Other	30 (5.2)	7/30 (23.3)	0.5 (0.2-1.3)		
Unreliable method of family planning used at baseline/reasons for non-use					
Use of unreliable method	245 (42.3)	92/245 (337.6)	1	<0.001	1
Breast feeding	141 (24.4)	111/141 (78.7)	6.0 (3.7-9.7)		5.0 (3.1-8.1)
Spent ≥2 years without getting pregnant	77 (13.3)	20/77 (26.0)	0.6 (0.3-1.1)		0.7 (0.4-1.2)
Side effects/fear	60 (10.4)	30/60 (50.0)	1.5 (0.9-2.6)		1.4 (0.8-2.4)
No access/money	31 (5.4)	25/31 (80.6)	7.2 (2.9-18.3)		6.8 (2.6-17.4)
Other	25 (4.3)	18/25 (72.0)	4.5 (1.8-11.1)		4.2 (1.7-10.7)

*uOR* unadjusted odds ratio, *CI* confidence interval, *LRT* likelihood ratio test, *aOR* adjusted odds ratio: factors adjusted for age, reasons for non-contraceptives use prior to enrolment, contraceptive use status at baseline, employment status and religion

**Fig. 2:**
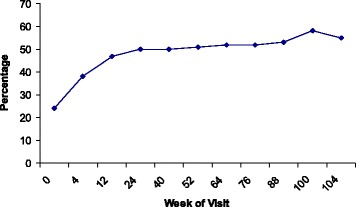
Proportion of women reporting use of hormonal contraception over time

As shown in Table 3, 296 (51.1%) women were defined as ‘new’ hormonal contraceptive users in terms of not using a reliable method at baseline and reporting use of a hormonal method during at least one follow-up visit. Of the new hormonal contraceptive users, 253 (85.5%) women started using DMPA, 41 (13.9%) oral pill and two (<1%) norplant. About two-thirds of new hormonal contraceptive users had reported not using any contraceptive method at enrolment (204; 68.9%), and the remainder had either switched from or supplemented condom use (85; 28.7%) or reported using another unreliable method (7; 2.4%). Figure 2 shows the proportion of women reporting hormonal contraceptive use at each of the extended behavioural visits throughout the trial. The graph illustrates that the majority of new hormonal contraceptive users started use within the first three months of follow-up and that hormonal contraceptive use was consistently over 50% from the six-month visit onwards.

Of the 283 women who did not start using a hormonal method of contraception, 8 opted for other reliable methods such as sterilisation (5) and the IUCD (3), while 250 women continued to report unreliable contraceptive methods such as condoms (242), and the natural rhythm method or withdrawal (8). Only twenty-five women never reported using any contraceptive method.

At enrolment, only 144 of the 807 (17.8%) women who had had sex in the previous 4 weeks reported dual use of a condom and a reliable contraceptive method at their last sex act. Of the 579 non-hormonal contraceptive users at baseline, 367 (63.4%) reported using a condom at their last sex act. Of the 296 new hormonal contraceptive users, 208 (70.3%) reported the use of a condom at the last sex act at every visit compared to 221/283 (78.1%) among the women who did not start using hormonal contraceptives.

Among women who reported not using any contraceptive method at enrolment, the main reasons included 141 (42.2%) reporting they were currently breast-feeding (perceived locally to prevent conception), 77 (23.0%) assumed they were infertile after at least 2 years of not conceiving, 60 (18.0%) had previous experience of side effects or a fear of using contraceptives, 31 (9.3%) reported lack of access or money for contraceptives, as well as 25 (7.5%) women reporting other reasons such as partner opposition or refusal, getting tired of using contraceptives and their partner wanting a child.

As shown in Table 3, factors that were significantly associated with hormonal contraceptive uptake at the 15% level in univariable analysis included age, religion, employment status, reported unreliable method of family planning at baseline, and reasons for not using a reliable method of contraception at baseline. After controlling for all factors in multivariable analysis, younger women (≤30 years) were more than twice as likely to have started using hormonal contraceptives, than older women (>30). Women were also significantly more likely to start using hormonal contraceptives if they had reported not using contraceptives at baseline due to a lack of access or money, breastfeeding or other reasons, compared to those who reported the use of other unreliable contraceptive methods.

### Sustained hormonal contraceptive use

Of the 296 new hormonal contraceptive users, 262 (88.5%) sustained use of the new hormonal method throughout the remainder of their follow-up. Most women reported use of hormonal methods for a median of 6 times (IQR 4–7) after initiation until end of follow-up. The remaining 34 (11.5%) switched between hormonal methods, with 29 starting on DMPA but switching to the oral pill, four starting on DMPA and switching to Norplant, and one starting with the oral pill and switching to Norplant. Women indicated bleeding as the main reason for switching from DMPA. After initiating hormonal contraceptive use, none of the 296 new contraceptive users reported not using hormonal contraceptives at a subsequent visit up to the end of their follow-up. There were no statistically significant differences between women who sustained the initial hormonal contraceptive method and those that switched methods in terms of age, religion, employment status or educational level (data not shown). Of the 197 women already using hormonal contraceptives at baseline, 193 (98.0%) sustained use of a hormonal method throughout follow-up.

## Discussion

In this study, we assessed contraceptive prevalence, uptake and sustained use among women enrolled in an HIV prevention trial in Masaka, who were in sero-discordant relationships. The baseline prevalence of reliable contraceptive use was low and similar to the national 2011 average among women of reproductive age in Uganda [3]. The specific use of hormonal contraceptives at baseline in this cohort was similar to the overall rate in the central region of Uganda [3] where the study was conducted. Contraceptive prevalence is consistently higher in Central and Western regions of Uganda [3] in comparison to the Northern and Eastern regions. A number of factors have been previously advanced for geographical variations in contraceptive use prevalence including community level cultural beliefs (such as value attached to children), the presence and quality of reproductive health services and accessibility in terms of transport routes [29]. The Northern and Eastern regions of Uganda were affected by a civil war for over two decades and this contributed to the breakdown of social services and community coping mechanisms [29].

In this study, the majority of women reporting reliable contraceptive use at baseline used DMPA, which is consistent with both national data and data from the central region of Uganda [3]. It was encouraging that contraceptive prevalence did not differ by age, educational status or religion. Nationally both younger age and lower educational status are associated with lower contraceptive use [3]. In this cohort unemployed women were significantly less likely to use contraceptives at baseline. In the national data, employment status is not reported yet women in the lowest wealth quintile had the lowest contraceptive prevalence [3], which is likely to explain the association observed with unemployment in our study.

The most striking finding from this study was that over half of all women who were not using reliable contraceptives at baseline started using hormonal contraceptives, mainly within the initial three months of the study. Uptake was significantly higher among younger women. Although our results do not suggest a significant age differentiation between women’s use of contraceptives at baseline, the uptake data suggests a substantial unmet need among this younger age group. The reasons that women reported for not using a reliable method of contraception at baseline were independently associated with hormonal contraceptives uptake. Women who reported a lack of access or a lack of money as being the main reason for non-use were over six times more likely to initiate hormonal contraceptive use than women using an unreliable method at baseline. Although contraceptives are freely available in Uganda, distance from health centres has been documented as a structural barrier to health services in Sub-Saharan Africa, including Uganda [30, 31]. This association between lack of access and low usage has been observed elsewhere in Uganda and Kenya [32, 33]. This finding, along with the correlation of lower use among unemployed women, highlights the need for either financial support for lower income women to be able to travel to existing health centres or for the expanded provision of more accessible family planning services.

In Uganda, and elsewhere, it is often believed that women cannot conceive when breastfeeding. Women who reported not using contraceptives at baseline due to breastfeeding were five-times more likely to initiate use during the study. This association has been observed in other parts of Africa [34] and highlights the need to disseminate accurate information about contraception and the need for better contraceptive messages and provision in ante-natal care settings [35].

Other reasons for non-contraceptive use reported at baseline such as partner opposition or refusal, women getting tired of using contraceptives and partners wanting a child, were also associated with high uptake of hormonal contraceptives during follow-up, although the numbers were low. In the Masaka centre we enrolled couples together and regularly provided couple counselling. Evidence from this centre suggests that generally couples jointly decided on use of the trial gel and women reported feeling supported by their partners to use the gel [36]. This evidence suggests that joint decision making or partner support could be an influencing factor in improving usage of hormonal contraceptives as well as gel.

It was encouraging to see very high levels of sustained use of hormonal methods among both women who reported using them at baseline and among new users. Uptake and sustained use was high in this cohort, and given the baseline contraceptive prevalence was similar to the local prevalence, it is likely that the study clinic filled a gap of provision in this community. However it is also likely that participation in the trial specifically motivated women to start and continue to use contraceptives. There is limited evidence on adherence and reasons for interruption and discontinuation, and further qualitative research is needed to help support appropriate uptake and sustained use in health care settings.

This study highlights the importance of offering hormonal contraceptives as a standard of care in clinical trials in order to reduce the risk of pregnancies requiring time off investigational product. This benefit has been illustrated in other HIV prevention trials as well [23, 37] However, there are concerns about the association between the use of hormonal contraceptives and an increased risk of HIV as observed in some studies [38–42] but not others [43–46]. Currently, the risks of withdrawing hormonal contraceptives outweigh the benefits in relation to increased risk of unwanted pregnancies and pregnancy complications [47–50]. The World Health Organisation has recommended continued use of hormonal methods until conclusive evidence of risk is available, with increased counselling for dual protection with condoms [51].

It was concerning to note that dual use of condoms and a reliable contraceptive method was very low at baseline among this cohort of HIV-negative women in sero-discordant relationships reportedly not wanting to get pregnant. However, baseline condom use for HIV prevention at the last sex act was high at 63%. Of particular importance is the fact that the increased uptake of hormonal contraception did not negatively impact on condom use, demonstrating the potential to improve dual method use. More effort is needed to promote dual contraceptive use especially in light of the World Health Organization (WHO) guidelines [51] recommending condom use along with hormonal methods to prevent possible increased risk of HIV acquisition.

Some of the limitations of this analysis are; we did not collect data on marital status, partner attitudes to contraceptive use, parity and area of residence all of which have been associated with contraceptive preference and use [3, 51, 52]. Furthermore, we did not collect data on the reasons for non-contraceptive use during follow up amidst free provision of contraceptives. Collection of such data could help inform future strategies aimed at improving contraceptive use during follow up.

The major strength of this study is that we assessed uptake in a study that provided contraceptives and where women were counselled on both the use and importance of adherence to dual contraceptive use. The contraceptive use data were collected by two independent investigators and cross checked at each visit.

## Conclusion

In a study with baseline contraceptive use similar to the general population, we saw a substantial uptake of hormonal contraceptives. The uptake of hormonal contraceptives did not displace the high use of condoms among sero-discordant couples, demonstrating the potential to improve the use of dual methods. The uptake was particularly pronounced among younger women and among women who couldn’t or didn’t think they needed to access contraceptives. The rate of sustained use was exceptionally high in a study with up to two years follow-up. This evidence highlights the benefit of promoting and providing hormonal contraceptives to trial participants where pregnancy could disrupt the use of investigational products. While contraceptive use has improved in Uganda, only a quarter of women of reproductive age report usage, unmet need remains high and dual method use low. As such this study also highlights the opportunities by which health care providers could fill gaps in provision including expanding access to low income and younger women, and enhancing accurate contraceptive messaging.

## Abbreviations

AIDS: Acquired Immunodeficiency Syndrome
aOR: Adjusted Odds Ratio
CI: Confidence Interval
DfID: UK Department for International Development
DMPA: Depot Medroxyprogesterone Acetate
HIV: Human Immunodeficiency Virus
IQR: Interquartile Range
IUCD: Intrauterine Contraceptive Device
LRT: Likelihood Ratio Test
MDP: Microbicides Development Programme
MRC: Medical Research Council
PrEP: Pre-Exposure Prophylaxis
UK: United Kingdom
uOR: Unadjusted Odds Ratio
UVRI: Uganda Virus Research Institute

## References

[1] WHO. http://who.int/mediacentre/factsheets/fs351/en/. Accessed 27 Feb 2017.

[2] Singh S, Darroch JE. Adding It Up: costs and benefits of contraceptive services estimates for 2012. New York: Guttmacher Institute and United Nations Population Fund (UNFPA); 2012. https://www.guttmacher.org/report/adding-it-costs-and-benefits-contraceptive-services-estimates-2012. Accessed 27 Feb 2017.

[3] Uganda Bureau of Statistics. Uganda Demographic and Health Survey 2011

[4] Jimmy R, Robert W, Bruno O, Allen K (2014). Modern contraceptive use among women in Uganda. An analysis of trend and petterns (1995–2011). Etude Popul Afr.

[5] Population reference Bureau. World population data sheet 2011.

[6] Guttmacher Institute. Contraception and Unintended Pregnancy in Uganda. Fact sheet 2013. http://www.guttmacher.org/pubs/FB-Contraception-and-unintended-pregnancy-in-Uganda.html. Accessed 20 Nov 2015.

[7] Khan Shane, Sarah E.K. Bradley, Joy Fishel, Vinod Mishra. Unmet Need and the Demand for Family Planning in Uganda: Further Analysis of the Uganda Demographic and Health Surveys, 1995–2006. Calverton, Maryland, USA: Macro International Inc.

[8] Corinne Dietiker, Cristin Gordon-Maclean, Lois K. Nantayi, Heidi Quinn, Thoai D. Ngo. Task sharing: Safety and acceptability of tubal ligation provision by trained clinical officers in rural Uganda. Research brief series (007) 2013.

[9] Uganda Ministry of Health (MOH). Press release of key results of the 2011 Uganda AIDS indicator survey

[10] Uganda AIDS Commission. National strategic plan for HIV&AIDS 2011/12 -2014/15

[11] Heena B, Makumbi F, Lutalo T, Sekasanvu J, Serwadda D, Wawer MJ (2014). Longitudinal study of correlates of modern contraceptive use and impact of HIV care programmes among HIV concordant and serodiscordant couples in Rakai, Uganda. J Fam Plann Reprod Health Care.

[12] Heffron R, Were E, Celum C, Mugo N, Ngure K, Kiarie J (2010). A prospective study of contraceptive use among African women in HIV-1 serodiscordant partnerships. Sex Transm Dis.

[13] Nayiga I, Semitala F.C, Matsiko N, Mutumba R, Nawavvu C, Namusobya J, et al. Low uptake of dual contraception methods and high prevalence of unwanted pregnancies among HIV-infected women in Uganda. In Proceedings of International AIDS society (IAS) Conference: Kuala Lumpur, Malaysia: 2013.

[14] Mumtaz Z, Slaymaker E, and Salway S. Condom use in Uganda and Zimbabwe: exploring the influence of gendered access to resources and couple-level dynamics. https://pdfs.semanticscholar.org/dfa6/3425bf94f45a8da96e73f06359e250e28efe.pdf. Accessed 27 Feb 2017.

[15] McCormack S, Ramjee G, Kamali A, Rees H, Crook AM, Gafos M (2010). PRO2000 vaginal gel for prevention of HIV-1 infection (microbicides development programme 301): a phase III, randomised, double-blind, parallel-group trial. Lancet.

[16] Van Damme L, Govinden R, Mirembe FM, Guedou F, Solomon S, Becker ML (2008). Lack of effectiveness of cellulose sulfate gel for the prevention of vaginal HIV transmission. N Engl J Med.

[17] Baeten JM, Donnell D, Ndase P, Mugo NR, Campbell JD, Wangisi J (2012). Antiretroviral prophylaxis for HIV prevention in heterosexual men and women. N Engl J Med.

[18] Kibengo FM, Ruzagira E, Katende D, Bwanika AN, Bahemuka U, Jessica EH (2013). Safety, Adherence and Acceptability of Intermittent Tenofovir/ Emtricitabine as HIV Pre-Exposure Prophylaxis (PrEP) among HIV-Uninfected Ugandan Volunteers Living in HIV-Serodiscordant Relationships: A Randomized, Clinical Trial. PLoS One.

[19] Marrazzo J, Ramjee G, Nair G et al. Pre-exposure prophylaxis for HIV in women: daily oral tenofovir, oral tenofovir/emtricitabine, or vaginal tenofovir gel in the VOICE study (MTN 003). 20th Conference on Retroviruses and Opportunistic Infections. Atlanta. March 3–6, 2013. Abstract #26LB.

[20] Abdool-Karim Q, Abdool-Karim SS, Frohlich JA, Grobler AC, Baxter C, Mansoor LE (2010). Effectiveness and safety of tenofovir gel, an antiretroviral microbicide, for the prevention of HIV infection in women. Science.

[21] Feldblum PJ, Adeiga A, Bakare R, Wevill S, Lendvay A, Obadaki F (2008). SAVVY vaginal gel (C31G) for prevention of HIV infection: a randomized controlled trial in Nigeria. PLoS One.

[22] Abdool-Karim SS, Richardson BA, Ramjee G, Hoffman IF, Chirenje ZM, Taha T (2011). Safety and effectiveness of BufferGel and 0.5% PRO2000 gel for the prevention of HIV infection in women. AIDS.

[23] Peterson L, Nanda K, Opoku BK, Ampofo WK, Owusu-Amoako M, Boakye AY (2007). SAVVY (C31G) gel for prevention of HIV infection in women: a phase III, double-blind, randomized, placebo-controlled trial in Ghana. PLoS One.

[24] Musekiwa A, Muchiri E, Manda SOM, Mwambi HG (2013). Pregnancy incidence and risk factors among women participating in vaginal microbicide trials for HIV prevention: systematic review and meta-analysis. PLoS One.

[25] Nunn A, McCormack S, Crook AM, Pool R, Rutterford C, Hayes R. Microbicides Development Programme: design of a phase III trial to measure the efficacy of the vaginal microbicide PRO 2000/5 for HIV prevention. Trials. 2009;10(1):99. 10.1186/1745-6215-10-99 PMC2774685 19860888

[26] Pool R, Montgomery CM, Morar NS, Mweemba O, Ssali A, Gafos M (2010). A mixed methods and triangulation model for increasing the accuracy of adherence and sexual behaviour data: the microbicides development programme. PLoS One.

[27] Jentsch U, Lunga P, Lacey C, Weber J, Cairns J, Gisela P (2012). The implementation and appraisal of a novel confirmatory HIV-1 testing algorithm in the Microbicides Development Programme 301 Trial (MDP301). PLoS One.

[28] Royston P, Ambler G, Sauerbrel W (1999). The use of fractional polynomials to model continuous risk variables in epidemiology. Int J Epidemiol.

[29] Asiimwe JB, Ndugga P, Mushomi J, Ntozi MJP (2014). Factors associated with modern contraceptive use among young and older women in Uganda; a comparative analysis. BMC Public Health.

[30] Ketende C, Gupta N, Bessinger R (2003). Facility-level reproductive health interventions and contraceptive use in Uganda. Int Fam Plan Perspect.

[31] Parkhurst JO, Rahman SA, Ssengooba F (2006). Overcoming access barriers for facility-based delivery in low-income settings: insights from bangladesh and Uganda. J Health Popul Nutr.

[32] Jaoko W G, Ogutu H, Wakasiaka S, Malogo R, Ndambuki R, Nyange J, et al. Pregnancy rates among female participants in phase I and Phase IIA AIDS Vaccine Clinical Trials in Kenya. East Afr Med J. 2009:86(9). 10.4314/eamj.v86i9.54165 21644413

[33] Okech CT, Wawire WN, Mburu KT. Contraceptive Use among Women of Reproductive Age in Kenya’s City Slums. Int J Bus Soc Sci. 2011:2(1).

[34] The tragic impact of unsafe abortion and inadequate access to contraception in Uganda. Center for Reproductive Rights, the International Women’s Human Rights Clinic (IWHRC), Georgetown Law Center Washington. The stakes are high report 2013. http://tbinternet.ohchr.org/Treaties/CESCR/Shared%20Documents/UGA/INT_CESCR_CSS_UGA_20279_E.pdf. Accessed 27 Feb 2017.

[35] Agbo HA, Ogbonna C, Okeahialam BN (2013). Factors related to the uptake of contraceptive in a rural community in Plateau State Nigeria: a cross-sectional community study. J Med Trop.

[36] Ashford L. Unmet need for family planning: Recent Trends and Their Implications for Programs. Population reference bureau Measure Communication. Policy brief 2003.

[37] Kawuma R: Role of men in a phase III vaginal microbicide trial in SW Uganda. In Proceedings of Microbicides Conference: 24–27. New Delhi, India: 2008.

[38] Crook AM, Ford D, Gafos M, Hayes R, Kamali A, Saida K (2014). Injectable and oral contraceptives and risk of HIV acquisition in women: an analysis of data from the MDP301 trial. Hum Reprod.

[39] Morrison CS, Skoler- Karpoff S, Kwok C, Chen PL, van de Wijgert J, Gehret- Plagianos M (2012). Hormonal contraception and the risk of HIV acquisition among women in South Africa. AIDS.

[40] Morrison CS, Chen PL, Kwok C, Richardson BA, Chipato T, Mugerwa R (2010). Hormonal contraception and HIV acquisition: reanalysis using marginal structural modeling. AIDS.

[41] Baeten JM, Benki S, Chohan V, Lavreys L, McClelland RS, Mandaliya K (2007). Hormonal contraceptive use, herpes simplex virus infection, and risk of HIV-1 acquisition among Kenyan women. AIDS.

[42] Heffron R, Donnell D, Rees H, Celum C, Mugo N, Were E (2012). Use of hormonal contraceptives and risk of HIV-1 transmission: a prospective cohort study. Lancet Infect Dis.

[43] Bulterys M, Chao A, Habimana P (1994). Incident HIV-1 infection in a cohort of young women in Butare, Rwanda. AIDS.

[44] Kapiga SH, Lyamuya EF, Lwihula GK, Hunter DJ (1998). The incidence of HIV infection among women using family planning methods in Dar es alaam, Tanzania. AIDS.

[45] Kiddugavu M, Makumbi F, Wawer MJ, Serwadda D, Sewankambo NK (2003). Hormonal contraceptive use and HIV-1 infection in a population -based cohort in Rakai, Uganda. AIDS.

[46] Kleinschmidt I, Rees H, Delany S, Smith D, Dinat N, Nkala B (2007). Injectable progestin contraceptive use and risk of HIV infection in a South African family planning cohort. Contraception.

[47] WHO (2010). Medical eligibility criteria for contraceptive use.

[48] Blish CA, Baeten JM (2011). Hormonal contraception and HIV-1 transmission. Am J Reprod Immunol.

[49] Curtis KM, Nanda K, Kapp N (2009). Safety of hormonal and intrauterine methods of contraception for women with HIV/AIDS: a systematic review. AIDS.

[50] Morrison CS, Turner AB, Jones LB (2009). Highly effective contraception and acquisition of HIV and other sexually transmitted infections. Best Pract Res Clin Obstet Gynaecol.

[51] WHO, Hormonal contraception and HIV**.** Technical statement 2012. 23586117

[52] Babirye S K. Uptake of modern contraception among youths (15–24) at community level in Busia district, Uganda. 2013 accessed at https://scholar.google.com/scholar?hl=en&q=Babirye+S+K.+Uptake+of+modern+contraception+among+youths+%2815%E2%80%9324%29+at+community+level+in+Busia+dist. Accessed 27 Feb 2017.

